# Septal Defects: Unveiling Sex-Based Disparities and Screening Challenges for Timely Intervention Through a Case Report and Systematic Literature Review

**DOI:** 10.7759/cureus.65752

**Published:** 2024-07-30

**Authors:** Elsy Rivera, Kathan Trivedi, George Cao

**Affiliations:** 1 Internal Medicine, Methodist Health System, Dallas, USA; 2 Internal Medicine, Methodist Dallas Medical Center, Dallas, USA

**Keywords:** sex-based differences, systematic review, adult case report, adult congenital heart disease, pulmonary hypertension, sinus venous atrial septal defects, atrial septal defects

## Abstract

Atrial septal defects (ASDs), comprising a significant portion of congenital cardiac anomalies, encompass a rarer and more diagnostically challenging subset known as sinus venosus ASDs (SVASDs). ASDs are more prevalent in females, and the prognosis for patients under 40 years of age is generally favorable with advancements in surgical and transcatheter interventions. However, undiagnosed ASDs in adults above 40 years old, especially females, often lead to severe complications, including pulmonary hypertension, atrial fibrillation, Eisenmenger syndrome, and a mortality rate exceeding 50%. Our detailed case study focuses on an obese 42-year-old Hispanic migrant female with chronic respiratory failure misattributed to pulmonary hypertension, resulting in the progression of complications from undiagnosed SVASD. Further investigation using contrast-enhanced transesophageal echocardiography (TEE) elucidated the correct diagnosis four years after her initial presentation. This report explores the potential factors contributing to the patient’s delayed diagnosis and development of advanced cardiac complications of pulmonary hypertension leading to Eisenmenger syndrome that precluded her from procedural intervention. Furthermore, this report pioneers the first thorough review of case reports in adults newly diagnosed with SVASD, revealing sex-based differences in complications.

## Introduction

Atrial septal defects (ASDs) are characterized by abnormal communication between the left atrium and systemic venous return. ASDs account for approximately 5%-12% of all congenital cardiac anomalies [1,2]. ASDs are more commonly diagnosed in females, with a female-to-male ratio of approximately 2:1 [2-4]. The prognosis for pediatric and adult patients with ASD under 40 years of age is generally favorable, especially with advancements in both surgical and transcatheter interventions, which allow for timely closure and reduce associated complications. In contrast, over half of adults above the age of 40 with untreated ASD develop sequelae of pulmonary hypertension (PH), atrial fibrillation, or pulmonary infections associated with a greater than 50% mortality rate [5,6].

Sinus venous ASDs (SVASDs) are rare, accounting for 5%-10% of ASDs [2]. Among various ASDs, SVASDs stand out for their inherent difficulty in being adequately visualized with transthoracic echocardiography (TTE) alone, particularly due to their posterior anatomical location at the cavoatrial junction [7]. Moreover, a 2005 study of outcomes in 115 patients who underwent surgical repair for SVASD found that a disproportionate number of patients who were older than 40 years were women, despite the equal prevalence of SVASD among the sexes [4,8]. This suggests that women may experience delays in diagnosis compared to men. In terms of outcomes, females with congenital heart disease are at higher risk of developing PH [9].

Similarly, in our encounter with a 42-year-old Hispanic female with a history of obesity and a prior diagnosis of chronic hypoxic respiratory failure attributed to PH, a screening TTE done four years before her admission did not reveal a causal diagnosis. Eventually, SVASD was demonstrated with a contrast-enhanced TEE. In this instance, the delayed diagnosis not only resulted in the progression to advanced Eisenmenger syndrome (ES) but also rendered her ineligible for surgical intervention. The underlying reasons for the delayed diagnosis as well as differences in outcomes in females remain unclear, prompting an exploration of potential factors in our detailed case study. Additionally, this report presents a comprehensive review of case reports among adults newly diagnosed with SVASD, with a specific focus on understanding the contributing factors to delayed diagnosis.

## Case presentation

A 42-year-old Hispanic female who migrated from Mexico with a past medical history of essential hypertension, chronic hypoxic respiratory failure on 2 L of oxygen via nasal cannula, and pulmonary hypertension presented to the hospital with a complaint of worsening dyspnea on exertion and lower extremity edema for three days. She was brought to the hospital via ambulance and was initially hypoxic with an oxygen saturation of 88% on 4 L of oxygen via nasal cannula. She had recently returned from a trip to Mexico. Furthermore, she endorsed paroxysmal nocturnal dyspnea as well as palpitations and denied any sick contacts. She was taking aspirin 325 mg. Further review of symptoms was negative. She had no history of smoking, drinking alcohol, or drug use. She denied a family history of premature coronary artery disease or premature cardiac death.

Of note, approximately four years before admission, she was diagnosed with PH on the initial right heart catheterization in the setting of chronic dyspnea following a TTE, which demonstrated a right ventricular systolic pressure (RVSP) of 94 mmHg. The TEE revealed associated right atrium and ventricle dilation but otherwise normal left ventricular morphology and ejection fraction. At that time, right heart catheterization revealed an elevated mean pulmonary arterial pressure (mPAP) of 50 mmHg (normal < 25 mmHg), elevated pulmonary vascular resistance of 480 dynes-sec/cm^5^ (normal < 240 dynes-sec/cm^5^), normal pulmonary arterial wedge pressure (PAWP) of 11 mmHg (normal < 15 mmHg), normal cardiac output, and normal left and right-sided filling pressures. There was no vasodilator response to adenosine. A ventilation-perfusion scan revealed subsegmental areas of decreased ventilation and perfusion corresponding to a low probability for pulmonary embolism by modified Prospective Investigation of Pulmonary Embolism Diagnosis (PIOPED) criteria. She was discharged home on losartan.

On presentation, the physical examination revealed the following findings: a heart rate of 69 beats per minute, a blood pressure of 124/82 mmHg, a respiratory rate of 24 breaths/minute, oxygen saturation of 82% on 6 L of oxygen via a nasal cannula, and a body mass index of 53.85 kg/m². Pertinent findings on physical exam included right ventricular heave, grade 2/6 holosystolic murmur heard best at the tricuspid area, elevated jugular venous pressure, and bilateral lower extremity edema.

**Figure 1:**
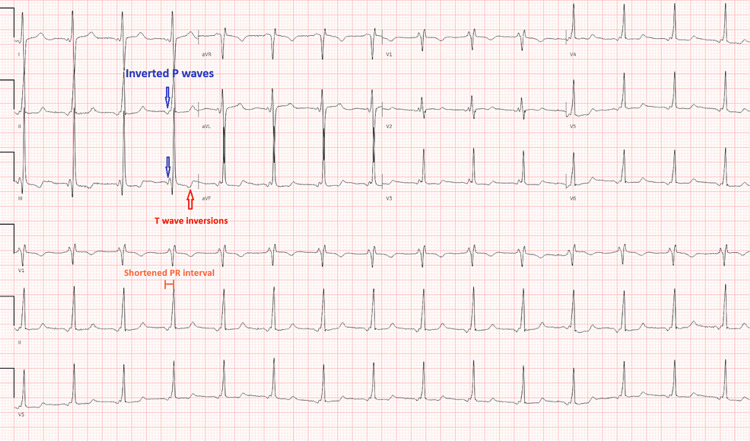
Electrocardiogram This electrocardiogram shows an unusual P axis and shortened PR interval, indicating a probable ectopic atrial rhythm. T-wave inversions are evident in the inferior leads. Nonspecific T-wave abnormalities are present and are worse in the anterior leads.

**Figure 2:**
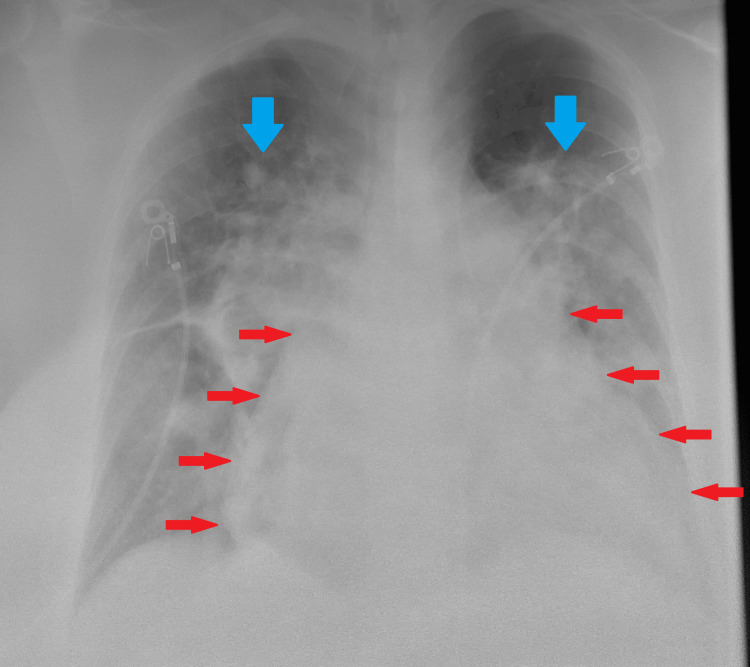
Anterior-posterior chest x-ray The chest radiograph in the anterior-posterior view of the patient reveals an enlarged cardiomediastinal silhouette (cardiac silhouette outlined by red arrows) and notable diffuse bilateral pulmonary opacities with a perihilar distribution (blue arrows).

An electrocardiogram showed normal sinus rhythm and prominent right axis deviation (Figure 1). A chest x-ray showed diffusely extensive bilateral pulmonary opacities with a perihilar distribution and an enlarged cardiomediastinal silhouette (Figure 2). A TTE with bubble study demonstrated a severely dilated right ventricle and right ventricular hypertrophy as well as shunting within three cardiac cycles.

**Figure 3:**
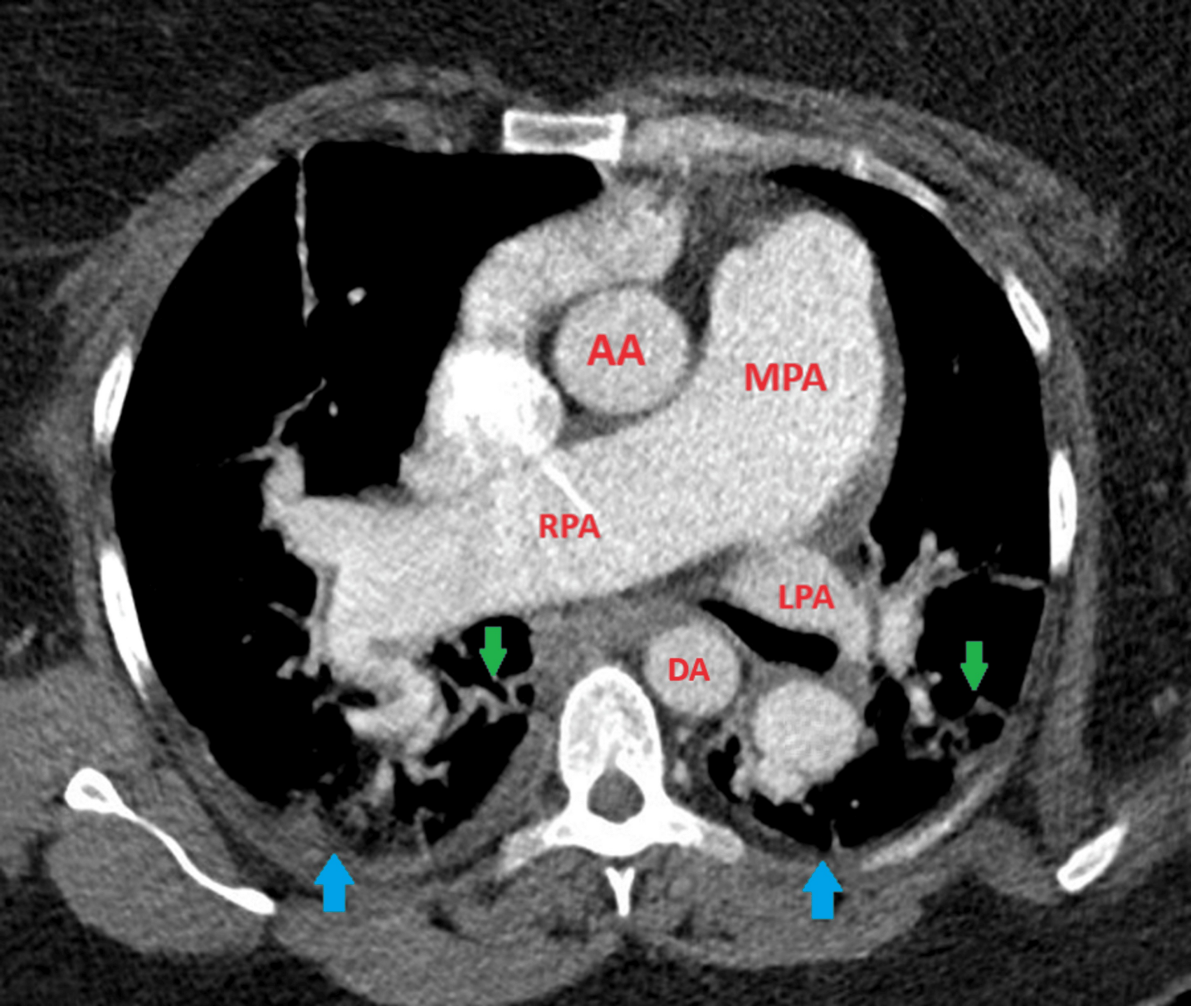
Computed tomography pulmonary angiography Computed tomography pulmonary angiography in the transverse plane reveals a patent pulmonary trunk, left pulmonary artery, and right pulmonary artery, without evidence of filling defects. There are notable bilateral infiltrates (green arrows) and small bilateral effusions (blue arrows). AA: Ascending aorta; DA: Descending aorta; LPA: Left pulmonary artery; MPA: Main pulmonary artery; RPA: Right pulmonary artery.

During her hospital course, she was given nebulized ipratropium-albuterol treatments every four hours, oral prednisone 40 mg daily, and intravenous furosemide 40 mg twice daily, resulting in an adequate urine output of >100 mL per hour. Over a day, her oxygen requirement increased to 15 L of high-flow nasal cannula, while her bilateral lower extremity edema continued to improve. Her RVSP was >85 mmHg, which was highly suggestive of severe PH of unknown etiology. This finding prompted an order of a CT angiography (CTA) of the chest to rule out chronic thromboembolic phenomenon as the cause of the PH. The CTA was negative for pulmonary embolism (Figure 3). Right heart catheterization was done to evaluate for WHO class I PH. The findings of this catheterization were PAWP of 19 mmHg, a mPAP of 58 mmHg, a pulmonary arterial resistance measured to 264 dynes-sec/cm^5^, and a very high cardiac output of 11 L/min.

**Figure 4:**
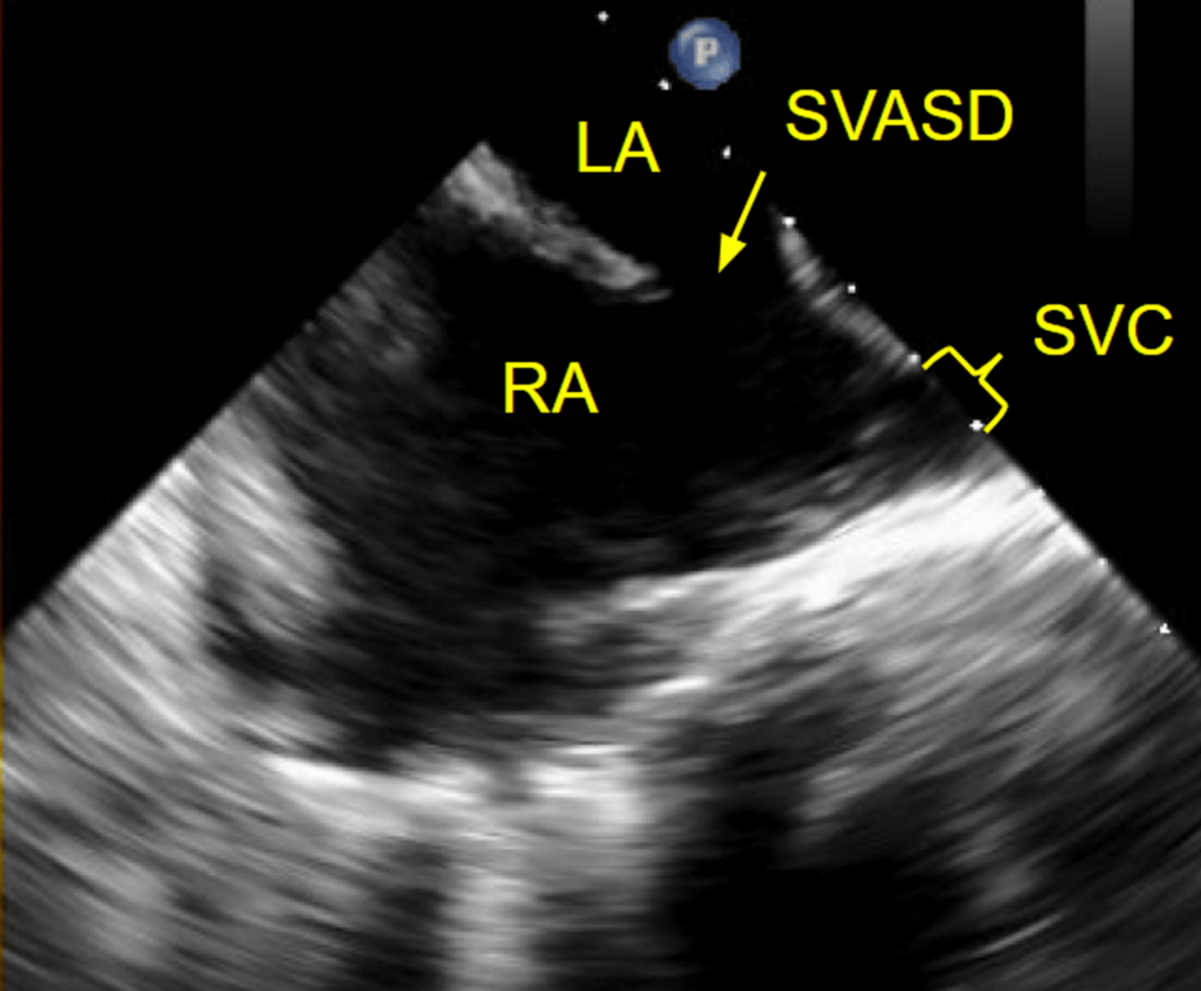
Transesophageal echocardiogram demonstrating sinus venosus atrial septal defect Transesophageal echocardiogram demonstrates sinus venosus atrial septal defect at the posterior aspect of the interatrial septum near the junction of the superior vena cava in conjunction with the right atrium. LA: Left atrium; RA: Right atrium; SVASD: Sinus venosus atrial septal defect; SVC: Superior vena cava.

A TEE was performed to further evaluate the interatrial septum based on the left-to-right shunt seen on the TTE bubble study. The bubble study with color Doppler 3D imaging demonstrated an ASD at the posterior aspect of the interatrial septum near the superior vena cava junction in conjunction with the right atrium, thus confirming the diagnosis of SVASD (Figure 4).

**Figure 5:**
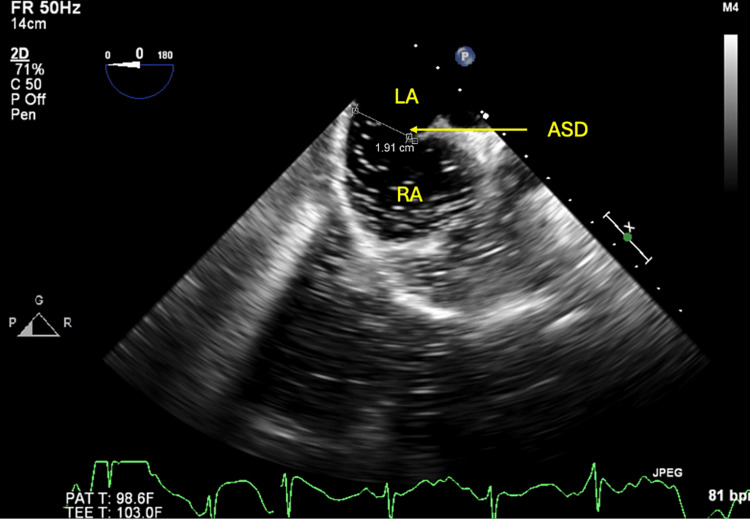
Transesophageal echocardiogram with agitated saline Transesophageal echocardiogram in the bicaval view demonstrates an atrial septal defect measuring approximately 1.91 cm. A left-to-right shunt is demonstrated at the atrial level through the usage of agitated saline contrast. ASD: Atrial septal defect; LA: Left atrium; RA: Right atrium.

Bi-directional shunting was demonstrated by agitated saline through an interatrial septal defect with a diameter of 1.91 cm (Figure 5). No intrapulmonary shunting was present, and no patent foramen ovale was visualized. Her RVSP was 75-80 mmHg, and a moderate tricuspid regurgitation was noted.

The patient was referred to cardiothoracic surgery for evaluation of SVASD repair. Surgical repair was deemed as a high mortality risk due to the advanced progression of cardiac changes, including the dilated right ventricle and elevated right pulmonary pressures. She was ultimately referred to a congenital cardiac surgeon. The patient was discharged on a stable oxygen requirement of 4 L of nasal cannula and oral furosemide and was ultimately lost to follow-up.

## Discussion

Methods

**Figure 6:**
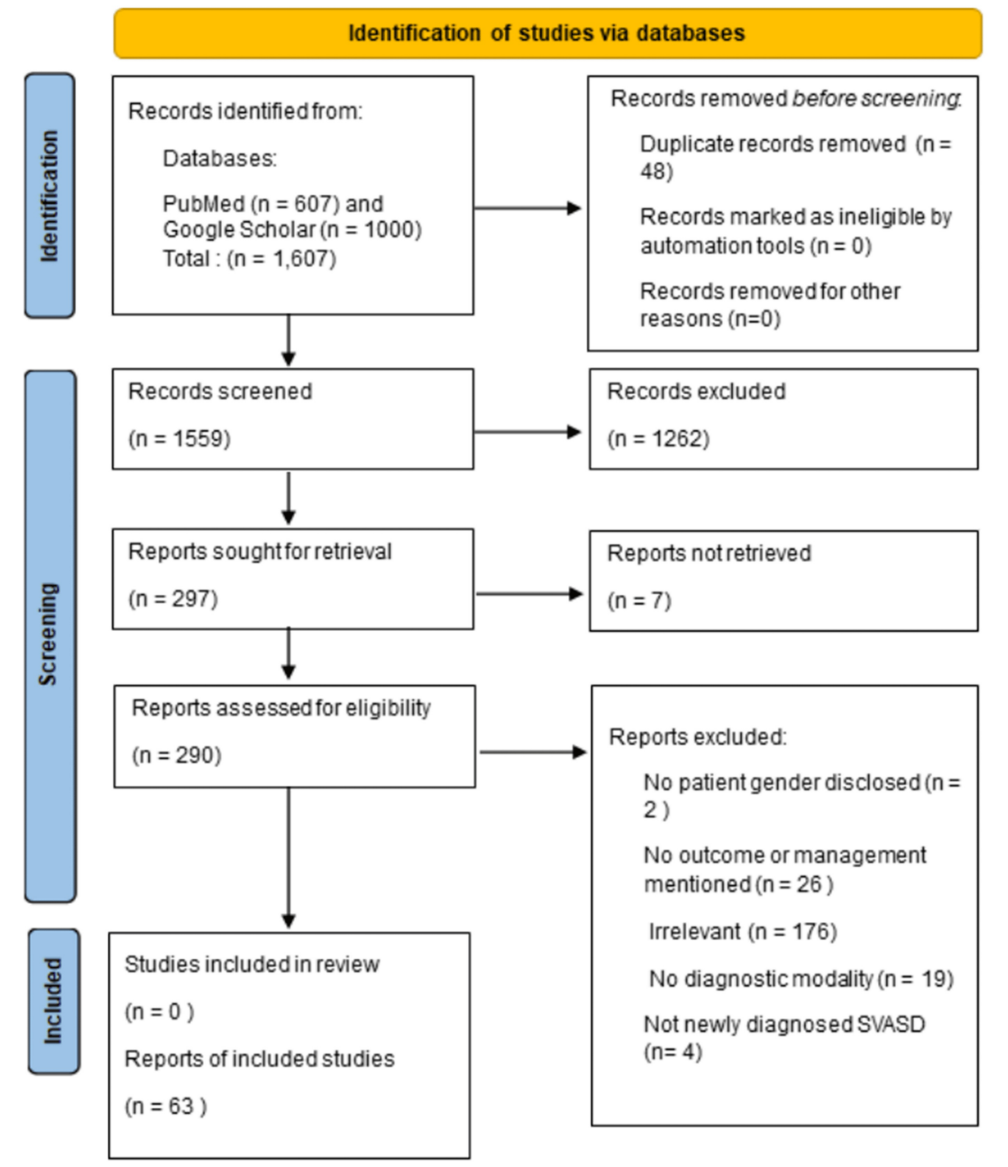
PRISMA flow diagram The image shows a systematic review of case reports of newly diagnosed sinus venosus atrial septal defects in adults with treatment or intervention outcomes using the Preferred Reporting Items for Systematic Reviews and Meta-Analyses (PRISMA) flow diagram [10,11]. SVASD: Sinus venous atrial septal defect.

A systematic PubMed and Google Scholar search was conducted to identify cases presenting with a new diagnosis of SVASD. The PubMed search term was “sinus venosus atrial septal defect” without limitations in terms of publication date. The Google Scholar search term was “‘sinus venosus atrial septal defect’ case report.” Full-text articles with abstracts were reviewed, and articles were excluded from further analysis using the following exclusion criteria: nonhuman studies, non-English language, pediatric cases, not case reports, and not SVASD (Figure 6).

In total, 297 articles were sought for retrieval. Out of these, seven articles could not be retrieved, and 227 studies were excluded for the following reasons: 176 for irrelevance, 26 for lacking outcome or management modality details, four for not newly diagnosed SVASD, 19 for no diagnostic modality mentioned, and two for not specifying participants’ gender. A focused cohort of 77 cases from 63 articles with documented SVASD presentations was assessed. Of note, there were five articles with 19 case reports as specified in Table 1.

**Table 1:** Case reports of newly diagnosed sinus venosus atrial septal defect in adults with treatment or intervention outcomes Single hyphens (-) indicate that information was not stated by the studies referenced. ASD: Atrial septal defect; Cath: Cardiac catheterization; CMR: Cardiac magnetic resonance; CT: Computed tomography; ES: Eisenmenger syndrome; F: Female; M: Male; PH: Pulmonary hypertension; Qp: Pulmonary flow; Qp: Systemic flow; RV: Right ventricle; RVSP: Right ventricular systolic pressure; TEE: Transesophageal echocardiogram; TTE: Transthoracic echocardiogram.

Patient	Age (years)	Sex (M/F)	Modality missed ASD	Modality visualized ASD	Pre-existing PH (Y/N)	Severity of RV dilation	RVSP (mmHg)	Shunt (Qp:Qs)	ES (Y/N)	Intervention	Reference
1	87	M	TTE	Cath	N	Present	-	2	-	Conservative	Bates, 1984 [12]
2	82	M	-	TTE	N	-	-	-	-	Declined	Davis et al., 2008 [13]
3	77	M	TTE	TEE, CT	N	-	-	-	-	Declined	Alhamshari et al., 2015 [14]
4	72	M	TTE	TEE, CT	Y	Moderate	50	-	-	Declined	Oe et al., 2024 [15]
5	68	M	TTE	TEE, CMR	Y	Severe	113	-	Y	Conservative	Dorosz et al., 2013 [16]
6	66	M	-	TTE, CT	Y	Severe	-	-	-	Conservative	Disha et al., 2014 [17]
7	66	M	TTE	TEE	N	Moderate	-	1.4	-	Surgical repair	Riahi et al., 2018 [18]
8	61	M	TTE	TEE	N	Severe	-	2.3	-	Surgical repair	
9	38	M	TTE	TEE	N	Severe	-	1.7	-	Surgical repair	
10	53	F	TTE	TEE	N	Severe	-	2.6	-	Surgical repair	
11	51	F	TTE	TEE	N	Severe	-	2.6	-	Surgical repair	
12	65	M	TTE	TEE, Cath	N	Severe	-	1.42	-	Surgical repair	Sugimori et al., 2021 [19]
13	77	F	TTE	CT, TEE	N	Severe	-	2.1	-	Surgical repair	
14	74	F	TTE	TEE, Cath	Y	Severe	-	1.81	-	Surgical repair	
15	63	M	TTE	Cath	N	Severe	-	3.1	-	Surgical repair	
16	71	F	TTE	Cath	N	-	-	-	-	Surgical repair	
17	63	M	TTE	CMR	N	Present	222	1.9	-	Transcatheter repair	Thakkar et al., 2018 [20]
18	63	M	TTE	CMR	N	Present	52	-	-	Surgical repair	Dudzinski et al., 2014 [21]
19	62	M	TTE	Cath	Y	Severe	55	-	-	Surgical repair	John et al., 2011 [22]
20	60	M	-	TTE, CMR	N	-	-	-	-	Conservative	Mousa et al., 2015 [23]
21	59	M	TTE	CMR	Y	Severe	35	2.2	-	Surgical repair	Donovan et al., 2015 [24]
22	59	M	TTE	TEE	N	Severe	50	2.8	-	Surgical repair	Schleiger et al., 2023 [25]
23	54	M	TTE	TEE	N	Severe	35	1.2	-	Surgical repair	
24	67	F	TTE	TEE	N	Severe	53	2.5	-	Surgical repair	
25	59	M	TTE	TEE	N	Present	65	-	-	Surgical repair	Pagel et al., 2016 [26]
26	53	M	TTE	CT	N	Severe	-	-	-	Surgical repair	Li et al., 2019 [27]
27	47	M	TTE	TEE, CT, Cath	N	Moderate	-	-	-	Surgical repair	Kulesh et al., 2021 [28]
28	46	M	-	TTE	N	Present	-	2.8	-	Surgical repair	Hidalgo et al., 2008 [29]
29	44	M	TTE	Cath, CMR	N	-	55	2.6	-	Conservative	D’Costa and Davidson, 1990 [30]
30	41	M	TTE	TEE, CT	N	Severe	-	-	-	Surgical repair	Ahn et al., 2012 [31]
31	41	M	TTE	CMR	Y	Severe	-	4.04	-	Surgical repair	De et al., 2014 [32]
32	35	M	-	TTE	N	Present	-	-	-	Surgical repair	Qiu et al., 2021 [33]
33	28	M	TTE, TEE	CMR	N	-	-	3	-	Surgical repair	
34	25	M	TTE	TEE	N	Severe	-	3	-	Surgical repair	
35	53	F	TTE	CMR	N	Severe	-	3	-	Surgical repair	
36	33	M	TTE	TEE	N	-	-	-	-	Declined	Pourkia et al., 2019 [34]
37	33	M	TTE	TEE	N	Present	-	-	-	Surgical repair	Betigeri et al., 2015 [35]
38	32	M	TTE	CMR	Y	Severe	-	5	-	Surgical repair	Oakley et al., 2014 [36]
39	29	M	TTE	CMR	N	-	-	-	-	Conservative	Pettersson et al., 2022 [37]
40	27	M	TTE	TEE	N	Severe	-	Negative	-	Surgical repair	Vodusek et al., 2019 [38]
41	26	M	-	TTE	N	Present	-	Increased	-	Surgical repair	Sharma et al., 2016 [39]
42	25	M	TTE	CMR	N	Moderate	22	2.1	-	Surgical repair	Ganigara et al., 2014 [40]
43	21	M	TTE	TEE	N	-	-	3	-	Surgical repair	Doven et al., 2001 [41]
44	19	M	TTE	TEE, Cath	N	-	-	-	-	Declined	Hsieh et al., 2007 [42]
45	85	F	TTE	CT	Y	Severe	57	4.7	-	Surgical Turn Down	Shimajiri et al., 2022 [43]
46	78	F	TTE	TEE, CT	N	-	1.6	1.6	-	Conservative	Topyla et al., 2017 [44]
47	76	F	TTE	CT	N	-	-	-	-	Surgical repair	Batteux et al., 2020 [45]
48	76	F	TTE	TEE, CT	Y	Severe	-	-	-	Surgical repair	Kim et al., 2016 [46]
49	73	F	-	TTE	N	Present	70	-	-	Transcatheter repair	Butera et al., 2019 [47]
50	72	F	TTE	TEE	N	Present	Normal	1.5	-	Conservative	Theodoropoulos et al., 2018 [48]
51	70	F	TTE	CMR	Y	Mild	-	2.65	-	Surgical repair	Singhal et al., 2018 [49]
52	65	F	TTE	Cath	N	-	-	2.7	-	Surgical repair	Meier et al., 2014 [50]
53	65	F	TTE	Cath	N	Present	-	2.2	-	Conservative	Koumi et al., 1989 [51]
54	63	F	TTE	TEE, Cath	N	Moderate	90	-	Y	Conservative	Leventopoulos et al., 2011 [52]
55	62	F	TTE	TEE	-	-	-	2.4	-	Declined	Saadat et al., 2010 [53]
56	61	F	TTE	CT	N	-	-	-	-	Surgical repair	Brendel et al., 1985 [54]
57	60	F	TTE	CMR	N	None	-	1.4	-	Conservative	Akpinar et al., 2013 [55]
58	57	F	TTE	TEE, CMR, CT, Cath	Y	Moderate	-	2.8	-	Surgical repair	Cetinarslan et al., 2018 [56]
59	57	F	TTE	TEE	N	Present	-	-	-	Surgical repair	Oh et al., 1988 [57]
60	53	F	TTE	TEE	Y	Severe	-	-	-	Surgical repair	Coon and Lang, 2006 [58]
61	42	F	TTE	TEE	Y	Moderate	-	-	-	Surgical repair	
62	50	F	TTE	CMR, CT	N	Present	-	-	-	Surgical repair	Vella et al., 2018 [59]
63	49	F	TTE	TEE, Cath	N	-	-	-	-	Surgical repair	Liang et al., 2021 [60]
64	47	F	TTE	CT	N	Moderate	-	-	-	Surgical repair	Baruteau et al., 2020 [61]
65	45	F	TTE	TEE	N	Present	-	2.3	-	Surgical repair	Moldovan et al., 2021 [62]
66	45	F	TTE, TEE	CMR	Y	Severe	40	2	-	Declined	Ghaemian et al., 2020 [63]
67	45	F	TTE	CMR, TEE, CT	N	Severe	Normal	1.7	-	Surgical repair	Mustapić et al., 2021 [64]
68	45	F	TTE	TEE, CT	N	Severe	-	-	-	Surgical repair	Kliger et al., 2012 [65]
69	45	F	-	TTE	Y	-	95	-	-	Surgical repair	Napoleone et al., 2012 [66]
70	43	F	TTE	CTA, TEE	Y	Severe	67	1.1	Y	Conservative	Anuwatworn et al., 2016 [67]
71	43	F	TTE	CT	N	-	-	-	-	Surgical repair	Alkhouli et al., 2018 [68]
72	34	F	TTE	CMR	Y	Severe	>60	2.5	-	Conservative	Rees et al., 2021 [69]
73	33	F	TTE	CMR	N	Present	27	2.6	-	Surgical repair	Kessel-Schaefer et al., 2006 [70]
74	31	F	TTE	CMR	Y	Moderate	47	1.9	-	Surgical repair	Gatzoulis and Giannakoulas, 2010 [71]
75	25	F	TTE	TEE, CMR	Y	Severe	140	-	Y	Conservative	Lovisolo et al., 2011 [72]
76	23	F	TTE	CMR	N	Mild	-	1.5	-	Surgical repair	O’Sullivan et al., 2016 [73]
77	23	F	TTE	TEE	N	Present	Moderate	-	-	Surgical repair	Leong et al., 2012 [74]

From this dataset, two investigators independently gathered information regarding patient age, sex, presence of a defect in the initial imaging modalities, RVSP, the presence of a previous diagnosis of PH, the severity of right ventricular dilation, the presence and severity of tricuspid regurgitation, the extent of the shunt (ratio of pulmonary blood flow (Qp) to systemic blood flow (Qs)), and the identification of ES. Information was also extrapolated regarding the confirmatory imaging modalities used to identify the shunts, including TTE, transesophageal echocardiography (TEE), cardiac magnetic resonance imaging (MRI), or cardiac computed tomography (CT). Finally, data on whether the subject was treated with conservative management or underwent surgical or catheter-based intervention were noted. Comparative analyses were used to summarize the clinical characteristics of the study population.

Results

Upon reviewing the 77 quality case reports, sufficient patient data was provided including confirmatory imaging, treatment modalities, and pertinent patient characteristics (Table 1). The data revealed that 34 out of 40 (85.0%) women and 24 out of 37 (64.9%) men were diagnosed above the age of 40. A mean age of 54.68 years for females and 49.43 years for males was calculated. Notably, eight out of 77 cases (10.4%) identified SVASD during the initial screening with TTE.

Moreover, our results highlighted a sex-based difference in the decision-making regarding surgery, with five out of 37 males (13.5%) compared to two out of 40 females (5.0%) declining procedural interventions. The mean RVSP of females was 62.5 mmHg compared to 53.2 mmHg in males. Prior diagnosis of PH was present in 13 out of 40 females (32.5%) and seven out of 37 males (18.9%).

Additionally, our findings indicated a severity difference in shunt characteristics between sexes. When calculating the percentage of right ventricular dilation in patients above 40 years of age, 15 out of 23 males (65.2%) had moderate or severe RV dilation compared to 17 out of 34 females (50.0%). Males exhibited a more severe shunt, as indicated by a mean Qp of 2.5 L/min/m^2^ compared to females with a mean Qp of 2.15 L/min/m^2^. Out of 40 females, four either developed ES or were surgically turned down compared to one male patient who developed ES.

Discussion

Studies have shown a comparatively worse outcome in adults newly diagnosed with SVASD who were older than 40 years [5]. This patient initially presented at 38 years old with milder cardiac symptoms, and her delayed SVASD diagnosis led to a significantly poorer outcome. In parallel, this literature review revealed that women are more likely to be diagnosed with SVASD above the age of 40 (85.0% of females) despite equal prevalence between sexes. Age plays a crucial role in treatment options as surgical repair after the age of 30 is associated with more complications, such as cerebral vascular accidents, arrhythmias, and worse New York Heart Association class functional status [75]. Older age of diagnosis correlates with increased complications and decreased survival, which may explain the increased incidence of ES in females. Only four other case reports of ES due to SVASD exist, with three being female. Notably, patients with ES have a survival rate of less than 50% after six years [76].

Major factors of undiagnosed congenital heart disease were present in this patient, including young age, migrant status, ethnic minority, early-onset right-sided heart failure, and PH. Congenital heart disease comprises approximately 10% of live births in Latin American countries, which is nearly 10 times higher than that of developed countries such as the United States [77,78]. Due to low screening rates in low-resource countries, congenital heart disease usually does not receive timely treatment. Ethnic minorities have increased barriers to care as a result of inadequate access, language barriers, educational disparities, and decreased health literacy [79]. These health inequities contribute to delays in seeking medical care as suspected in our case and another case of SVASD complicated by ES in a Hispanic female [67]. Overall, younger females from countries with low rates of congenital heart screening presenting with right heart failure and early-onset dyspnea should trigger a broader differential diagnosis to include consideration of undiagnosed congenital heart disease.

This study underscored the importance of considering alternative diagnoses in patients with specific demographics due to the limitations of routine screening TTE, which missed this patient’s SVASD. The literature review confirmed the limitations of echocardiography in diagnosing SVASD where only 10.4% of cases were visualized using screening TTE. Contrast enhancement was not specified in any reports. Given the posterior anatomic location of SVASD, two-dimensional TTE has a false negative detection rate of more than 50%. As such, confirmatory testing with cardiac CT or cardiac MRI has been shown to detect SVASD with a higher rate of visualization [80]. Alternatively, a TTE with agitated saline (bubble study) is a non-invasive and cost-effective screening method for ASD. If the initial TTE noted abnormal results in a contrast-enhanced study, further investigation for the origin of the shunt could have significantly altered the prognosis of this patient.

Other sex-based differences in congenital heart disease were noted in this review. Women presented with more advanced stages of disease characterized by a higher RVSP than their male counterparts. On the other hand, RV dilation and shunt were more severe in males above the age of 40. It is unclear why males were more likely to have increased right ventricular dilation compared to women, especially those above 40 years. Feasibly, women may have more physiologic reserve to compensate for their chronic elevated pulmonary pressure, which may underestimate the disease severity. Furthermore, women with congenital heart disease, including SVASD, have a higher incidence of PH. Hormonal factors and pregnancy are postulated to account for higher rates of PH in females [9]. Females in this review were 1.7 times more likely to have a prior diagnosis of PH. Higher rates of PH may lead to misattribution of symptoms, premature termination of workup, and delayed diagnosis of congenital heart disease in women, as noted in this case report.

This comprehensive review evaluated qualitative data and factors contributing to delayed diagnoses of SVASD in patients. The patient in this study faced significant barriers to accessing healthcare because of underlying factors such as the lack of familiarity with the healthcare system, poor health literacy, language barriers, lack of documentation status, and financial constraints. Investigating these factors was of interest in conducting the first systematic review of case reports of newly diagnosed adults with SVASD. However, socioeconomic factors were not extensively documented in any reviewed case report.

## Conclusions

Findings from our encounter with the presented patient inspired the extensive systematic review, which underscores the critical importance of timely diagnosis in patients with SVASDs. If SVASD had been identified earlier, before the manifestation of ES, there could have been an opportunity for proactive management and intervention. The potential delay in diagnosis, compounded by the advanced cardiac changes observed in this patient, emphasizes the necessity for heightened clinical suspicion, comprehensive diagnostic approaches, and the incorporation of contrast-assisted screening echocardiography. Recognizing SVASD in the early stages is pivotal as delayed diagnosis may render intervention less effective or even unfeasible, highlighting the need for increased vigilance in the assessment of patients at high risk for undiagnosed congenital heart disease. This systematic literature review of case reports in adults with newly diagnosed SVASD revealed the need for a further investigation of age and gender disparity in congenital heart disease.
